# mSphere of Influence: Predicting the evolution of pathogen populations

**DOI:** 10.1128/msphere.00432-24

**Published:** 2024-07-26

**Authors:** Tatum D. Mortimer

**Affiliations:** 1 Department of Population Health, College of Veterinary Medicine, University of Georgia, Athens, Georgia, USA; University of Georgia, Athens, Georgia, USA

**Keywords:** pathogen evolution, prediction, genomics

## Abstract

Tatum D. Mortimer works in the field of pathogen population genomics and evolution. In this mSphere of Influence article, she reflects on how “Frequency-dependent selection can forecast evolution in *Streptococcus pneumoniae*” by Azarian et al. and “Contingency, repeatability, and predictability in the evolution of a prokaryotic pangenome” by Beavan et al. made an impact on her by highlighting the ways in which genomic data can be used to predict pathogen evolution.

## COMMENTARY

As antimicrobial resistance continues to emerge and spread among bacterial pathogens, accurate prediction of pathogen evolution would be an invaluable tool to assess the potential impact of new treatments and public health interventions on microbial populations or identify strains at risk for evolving new traits. Large whole genome sequencing efforts have enabled a detailed view of variation in bacterial populations, and there is a growing body of literature seeking to uncover the evolutionary mechanisms maintaining this diversity. However, with complex patterns of diversity and interacting selection pressures, predicting the evolution of natural populations of pathogenic bacteria is not a trivial task. In “Frequency-dependent selection can forecast evolution in *Streptococcus pneumoniae*” by Azarian et al. (1) and “Contingency, repeatability, and predictability in the evolution of a prokaryotic pangenome” by Beavan et al. (2), models based on accessory gene presence and absence are used to predict the prevalence of strains within a population and the co-occurrence of genes within the pangenome, respectively.

Recent work has demonstrated that negative frequency dependent selection (NFDS), where the fitness of an allele is dependent on its frequency in the population, impacts the evolution of multiple pathogens (3, 4). In *Streptococcus pneumoniae*, genomes that are part of the same strain defined on the basis of the core genome also encode similar accessory gene content and serotype. Despite variation in the frequency of these strains across geographic regions, the frequency of accessory genes is remarkably similar (3). Perturbations to *S. pneumoniae* populations can have unexpected impacts on the strain distribution, and as strains are removed from the population through interventions like vaccination, the post-intervention strain distribution cannot be accurately predicted from the pre-intervention strain distribution alone. Azarian et al. sought to improve the prediction of post-vaccine strain distributions using models based on NFDS. Using a deterministic model, they were able to estimate the fitness of strains based on accessory gene content. Additionally, they used quadratic programming and the frequency of accessory genes in the pre-vaccine population to predict the strain composition of the *S. pneumoniae* population in the southwest United States after the population had regained equilibrium following perturbation by vaccination. Subsequent work demonstrated that modification to these NFDS-based models could still predict post-vaccination strain distributions using non-representative samples from invasive infections (5), allowing these models to be used in conjunction with data from genomic surveillance programs that were not specifically designed for prediction.

NFDS is not the only force impacting the distribution of accessory genes. Genes can be advantageous in some environments or genetic backgrounds but not others (6). Beavan et al. used a random forest model trained on the pangenome of publicly available *Escherichia coli* complete genomes to identify gene pairs where presence or absence was correlated. Importantly, they considered the impact of population structure by focusing on accessory genes distributed across the phylogeny rather than lineage-specific genes. They found that the distribution of a subset of the *E. coli* pangenome was predictable across multiple runs of the model, and these genes represented diverse biological functions. Physical linkage appeared to influence co-occurrence for many of these genes; however, some gene pairs were not physically linked or displayed an avoidant relationship where they were never found in the same genomes. For example, the absence of *pac*, a penicillin acylase, can be predicted by the presence of *symE*, a component of a type I toxin-antitoxin system. The number of gene co-occurrence pairs identified using this model was sensitive to the sample size used for training. As the accuracy of long-read sequencing technologies continues to improve and the number of high-quality genomes increase, the predictive power of these models may improve. By understanding interactions between accessory genes and their genetic backgrounds, we may be able to predict which lineages are at risk for acquiring genes of interest, like those associated with virulence or antimicrobial resistance.

Although sequencing-related costs have substantially decreased over the last decade, genomic surveillance programs still represent a substantial investment for public health agencies and diagnostic laboratories. The work by Azarian et al. and Beavan et al. highlight the potential contribution of genomics to infectious disease prediction and forecasting. These predictive models have primarily been focused on variation in the accessory genome. However, gene presence or absence is only one of the ways that diversity is maintained in bacterial populations, and some of the pathogens that I have studied during my scientific career, including *Mycobacterium tuberculosis* and *Neisseria gonorrhoeae,* have limited accessory genomes compared with species like *S. pneumoniae* and *E. coli*. These studies have inspired me to reflect on how we might apply similar approaches to predict the dynamics of other types of variation in bacterial populations. Can we predict changes in strain prevalence or the acquisition of new alleles of core or accessory genes on the basis of single nucleotide polymorphisms, insertions, and deletions? Results from Azarian et al. and others (1, 3) suggest that, while they do not have the same predictive power as accessory genes, alleles in the *S. pneumoniae* core genome are also subject to NFDS. In addition to generating predictive models, identification of interactions between alleles may provide new insights into the biology of these important pathogens.
